# Revisiting Biomarkers of Total-Body and Partial-Body Exposure in a Baboon Model of Irradiation

**DOI:** 10.1371/journal.pone.0132194

**Published:** 2015-07-15

**Authors:** Marco Valente, Josiane Denis, Nancy Grenier, Philippe Arvers, Barbara Foucher, François Desangles, Patrick Martigne, Hervé Chaussard, Michel Drouet, Michael Abend, Francis Hérodin

**Affiliations:** 1 Département des Effets Biologiques des Rayonnements (EBR), Institut de Recherche Biomédicale des Armées (IRBA), Brétigny-sur-Orge, France; 2 Service des Laboratoires, Hôpital d'Instruction des Armées Desgenettes, Lyon, France; 3 Bundeswehr Institute of Radiobiology, Munich, Germany; Georgetown University, UNITED STATES

## Abstract

In case of a mass casualty radiation event, there is a need to distinguish total-body irradiation (TBI) and partial-body irradiation (PBI) to concentrate overwhelmed medical resources to the individuals that would develop an acute radiation syndrome (ARS) and need hematologic support (i.e., mostly TBI victims). To improve the identification and medical care of TBI versus PBI individuals, reliable biomarkers of exposure could be very useful. To investigate this issue, pairs of baboons (n = 18) were exposed to different situations of TBI and PBI corresponding to an equivalent of either 5 Gy ^60^Co gamma irradiation (5 Gy TBI; 7.5 Gy left hemibody/2.5 right hemibody TBI; 5.55 Gy 90% PBI; 6.25 Gy 80% PBI; 10 Gy 50% PBI, 15 Gy 30% PBI) or 2.5 Gy (2.5 Gy TBI; 5 Gy 50% PBI). More than fifty parameters were evaluated before and after irradiation at several time points up to 200 days. A partial least square discriminant analysis showed a good distinction of TBI from PBI situations that were equivalent to 5 Gy. Furthermore, all the animals were pooled in two groups, TBI (n = 6) and PBI (n = 12), for comparison using a logistic regression and a non parametric statistical test. Nine plasmatic biochemical markers and most of hematological parameters turned out to discriminate between TBI and PBI animals during the prodromal phase and the manifest illness phase. The most significant biomarkers were aspartate aminotransferase, creatine kinase, lactico dehydrogenase, urea, Flt3-ligand, iron, C-reactive protein, absolute neutrophil count and neutrophil-to-lymphocyte ratio for the early period, and Flt3-ligand, iron, platelet count, hemoglobin, monocyte count, absolute neutrophil count and neutrophil-to-lymphocyte ratio for the ARS phase. These results suggest that heterogeneity could be distinguished within a range of 2.5 to 5 Gy TBI.

## Introduction

Exposure to a nuclear or radiological (NR) event would lead to a heterogeneous population, with different radiation doses and degrees of shielding. The effectiveness of the medical care that follows depends on early triage and diagnosis of the victims to separate the “worried well” from the irradiated victims, especially in case of a large scale event. Moreover, the discrimination between TBI and PBI patients is of vital importance as they will have a different clinical outcome. Medical management relies on physical, clinical and biological dosimetry. In accidental or malevolent situations, physical dosimeters would not be worn. Clinical signs and symptoms would therefore be the main tools for early triage [1]. Thus, there is a need of easy-to-use biomarkers to improve medical management at the different phases of clinical evolution.

The dicentric chromosome assay (DIC) is the gold standard for radiation dose estimate which is based on the scoring of these unstable aberrations in peripheral blood lymphocytes. In addition to the dicentric frequency, the distribution per cell can also be used to identify heterogeneous exposures. However, there is no linear relationship between the respective volumes of peripheral blood lymphocytes and bone marrow exposed. Moreover, DIC cannot account for radiation-induced tissue functional effects depending on the dose delivered to a given organ, the irradiated volume and the dose distribution. Indeed there are regional variations in clinical consequences for each organ [2]. Furthermore, DIC is not a fieldable technique and results are not available until after 3 days following blood sampling [3]. The slope of peripheral blood (PB) lymphocyte count and the levels of plasmatic parameters such as C-reactive protein (CRP), interleukin 6 (IL-6), serum amyloid protein (SAA) and amylase are relevant early bio-indicators of exposure for TBI [4–6]. Nevertheless, the reliability of these markers to discriminate between TBI and PBI has not yet been established.

Here, we assessed the feasibility of using several biomarkers to identify PBI versus TBI at different time windows after exposure. The baboon model was chosen for its genetic and physiological proximity to humans and its size, which allowed us to take in consideration the impact of corpulence in dose distribution [7]. Moreover, unilateral irradiation was chosen to account for accidental exposure. We compared six partial-body exposures and three whole-body exposures that were equivalent to either 2.5 Gy or 5 Gy TBI. At this dose range baboons develop an acute radiation syndrome for mainly global exposures [8]. Therefore this is a case where the information of TBI versus PBI would be of vital importance for medical care. In addition to DIC assessment, we investigated a large panel of biomarkers that could be evaluated with field capabilities following a nuclear or radiological event, such as absolute neutrophil count (ANC), absolute lymphocyte count (ALC), neutrophil-to-lymphocyte ratio (NLR), monocyte count (MONO), platelet count (PLT), hemoglobin (Hb), aspartate aminotransferase (AST), creatine kinase (CK), lactico dehydrogenase (LDH), urea, Flt-3 Ligand, iron, and CRP level. The course of exploration covered not only the period of diagnosis but also the manifest illness phase when patients’ clinical review could be required for treatment decision making.

## Materials and Methods

### Animals

The twenty-one adult male baboons weighing 23.2 ±5.3 kg used in this work were housed in the nonhuman primate facility of the French Army Biomedical Research Institute (IRBA), as described in previous works [8–9]. The experiment was approved by the French Army Animal Ethics Committee (No 2010/12.0). All baboons were treated in compliance with the European legislation related to animal care and protection. Specifically, the veterinary surgeon in charge of animal welfare at IRBA’s animal facility fulfilled a mission of council and inspection to ensure that nonhuman primates were provided supportive care in ways that minimize fear, pain, stress, and suffering. Accordingly, two baboons showing signs of pain received buprenorphine (Vetergesic Multidose, Sogeval, Sheriff Hutton, UK). Furthermore, animals were transfused with fresh, whole blood irradiated with a 20 Gy gamma dose when PLT was lower than 20 x 10^9^/L with obvious signs of petechiae or the hematocrit less than 20%. Ampicillin (50 mg/kg/day) and gentamycin (1.5 mg/kg/day) were provided during neutropenia (ANC lower than 0.5 x 10^9^/L) and cefalexin (1 mg/kg/ day for 3 days) during prolonged febrile periods above 39°C.

### Irradiation

The animals were anesthetized and then exposed to a ^60^Co source [8–9]. Two baboons were exposed to 5 Gy TBI and two other to 2.5 Gy TBI. For 10 Gy 50% PBI (n = 2) and 5 Gy 50% PBI (n = 2) which are vertical left hemi-body irradiations, the right side of the body was shielded using a 20 cm thick lead screen. The same shielding device was used to perform the 7.5 Gy / 2.5 Gy (left and right hemi-bodies respectively, n = 2) TBI model. For the four other PBI situations, the lead screen was placed so as to shield either the left leg (5.55 Gy 90% PBI, n = 2), the two legs up to femoral heads (6.25 Gy 80% PBI, n = 3), the head, the cervical vertebrae and the neck (6.25 Gy 80% PBI, n = 1) or to expose the head and the two arms only (15 Gy 30% PBI, n = 2). Two dose rates were used (8 cGy/min for 5 Gy TBI and 5 Gy 50% PBI, and 32 cGy/min for all other situations) because the Cobalt 60 source was changed during this study. Moreover, to warrant the same homogeneous radiation field whatever the dose rate, all baboons were irradiated at the same distance from the source. Consequently radiation exposures lasted between 8 min and 62 min. The mid-line tissue (right anterior iliac crest) dose in air was measured with ionization chamber. Delivered doses were controlled by alumina powder thermoluminescent dosimeters placed on different cutaneous areas (thorax, thoracic and lumbar vertebrae, head, tibia, femur, femoral head). Moreover, in order to assess the impact of frequent blood sampling on hematologic status, a group of three age-matched baboons was used as sham-irradiated individuals that were handled as irradiated animals (in terms of anesthesia, handling in restraint chair). The same blood volumes were then collected over the days following irradiation.

### Clinical dosimetry

Time to onset, severity and duration of 7 clinical signs were recorded: vomiting, erythema, diarrhea, petechiae, hair loss, fever, and body weight loss. Each symptom was quantified (i.e. score 0, 1, 2 or 3) depending on presence and severity. Measurements were made at 22 different time points: before irradiation and at 1hour (h), 6h, 12h, 1day (d), 2d, 3d, 4d, 5d, 7d, 10d, 14d, 17d, 21d, 24d, 28d, 35d, 42d, 60d, 90d, 120d and 200d after irradiation.

### Biologic parameters

Nine hematological parameters were performed on a Pentra 120 analyzer (ABX, Montpellier, France) and using May-Grünwald Giemsa stain (Sigma, Saint-Quentin Fallavier, France): ANC, ALC, MONO, red blood cells (RBC), PLT, Hb level, hematocrite and mean corpuscular volume (MCV). Additionally, the neutrophil-to-lymphocyte ratio (NLR) was calculated and also used as a parameter. Measurements were made at the same 22 time points used for clinical dosimetry.

The following 7 coagulation and fibrinolysis factors were evaluated: activated partial thromboplastin time, prothrombin time, thrombin time, fibrinogen level, factor V, Fibrin d-dimer and monomer (DiagnosticaStago, Asnières, France). Furthermore, the following 26 plasmatic biomarkers: albumine, total protein, chloride, sodium, potassium, lactate, alanine aminotransferase (ALT), AST, amylase, CK, LDH, alkaline phosphatise (ALP), cholesterol, triglycerides, urea, creatinine, glucose, citrullin, EPO, Flt-3 Ligand, Iron, C3c, C-reactive protein, haptoglobin, orosomucoid, transferring were measured using Hitachi 912 Analyzer (Roche Diagnostics, Meylan, France). Citrulline was assessed by high-performance liquid chromatography (Biomnis, Lyon, France). EPO (Erythropoietin) and Flt-3 Ligand were assessed using enzyme linked immunoassay (R & D Systems, Abingdon, UK). Most coagulation, fibrinolysis factors and plasmatic biomarkers were measured at 17 different time points: before irradiation and at 1d, 2d, 3d, 4d, 5d, 7d, 10d, 14d, 21d, 24d, 28d, 42d, 60d, 90d, 120d and 200d after irradiation.

Additionally, hematopoietic bone marrow (BM) colony-forming units from exposed and shielded humerus were evaluated before irradiation and at 1d, 42d and 200d after TBI and PBI (as described in [10]). These results were not included in the 51 parameters of the global statistical analysis as the intrusive type of sampling would not be practical in the field.

### Dicentric chromosome assay

The anubis baboon has a diploid chromosome number of 42 with a minute akrocentric Y chromosome. Dicentrics frequency and distribution per cell were evaluated (2 parameters) before exposure and at 1h, 6h, 1d, 28d and 200d after irradiation (6 time points). Dicentric frequency was determined for at least 250 lymphocytes (or 100 dicentrics) per sample. The u-value of the dicentric distribution was calculated to determine if the exposure was inhomogeneous (PBI) and the Dolphin method [11] was used to estimate the irradiated fraction and dose. The estimated doses were calculated with the program CABAS [12]. A curve was previously established based on the *in vitro* exposure (0, 0.25, 0.5, 1, 2, 2.5, 4 and 5 Gy) of blood from two animals.

### Statistics methodology

The analysis was based on three independent statistical approaches. A first general analysis method was carried out on all the data of all 5 Gy equivalent baboons to validate the model. In the following approaches, the analysis of each biomarker was done by comparing the combined PBI data with the combined TBI data.

A soft independent modelling of class analogy using a non-linear iterative partial least square discriminant analysis (PLSDA) using SIMCA Version11.5 (Umetrics, Umea, Sweden) was carried out on 11331 (of the 12950 expected) individual values from fourteen TBI and PBI baboons equivalent to a 5 Gy global exposure. The expected values are the product of 51 biological and clinical markers multiplied by their experimental times (925 variables), multiplied by 14 baboons. The 1619 missing values correspond to data that could not be recovered for technical or logistical reasons that led to insufficient quality or quantity of sample. This first general analysis method allows the classification of individuals based on a great number of samples and was used to validate the experimental radiation model.

As a second approach, all the animals were pooled in two groups, TBI (n = 6) and PBI (n = 12) for comparison employing univariate logistic regression analysis (SAS Version 9.1.3, Cary NC, USA). This regression was performed to assess odds-ratio (OR) which quantify the association probability between a dichotomic variable (TBI vs PBI exposure) and each of the independent clinical or biological factors susceptible to influence it (14849 values of the 16650 expected for 18 animals). Corresponding 95% confidence intervals, p-values (chisq) were generated. We also calculated the area under a ROC (receiver-operator characteristic) curve providing a reasonable indication of overall diagnostic accuracy. ROC areas of 1.0 indicate complete agreement between the predictive model and known exposure status and thus a clear distinction between partial and total body irradiation. Statistical significant frequency differences in e.g. vomiting or diarrhea were calculated using chi-square statistics. All calculations were performed using SAS (release 9.1.3, Cary NC, USA).

Thirdly the Mann-Whitney non parametric rank test was carried out on all data to analyse the potential of each clinical or biological parameter to discriminate between the combined TBI data and the combined PBI results for a given time independently of pre-irradiation values.

The comparison of pairs of animals with the same exposure was only preformed for the DIC assay as this marker is already the reference for the classification of exposure victims.

## Results

### Physical dosimetry

Body dosimetry showed an average gradient of dose distribution front-to-back equal to 2.9 for both TBI and the exposed side of PBI. For PBI, the dose delivered to shielded areas was roughly 6% of that delivered to exposed areas.

### Clinical and biological dosimetry

With respect to clinical parameters, petechiae were observed around 15d for TBI baboons only. Furthermore, regarding animals for which cutaneous areas were exposed to doses equal to or higher than 7.5 Gy (whatever TBI or PBI situation) erythema was observed at 2d to 6d and hair loss from 21d.

**Fig 1:**
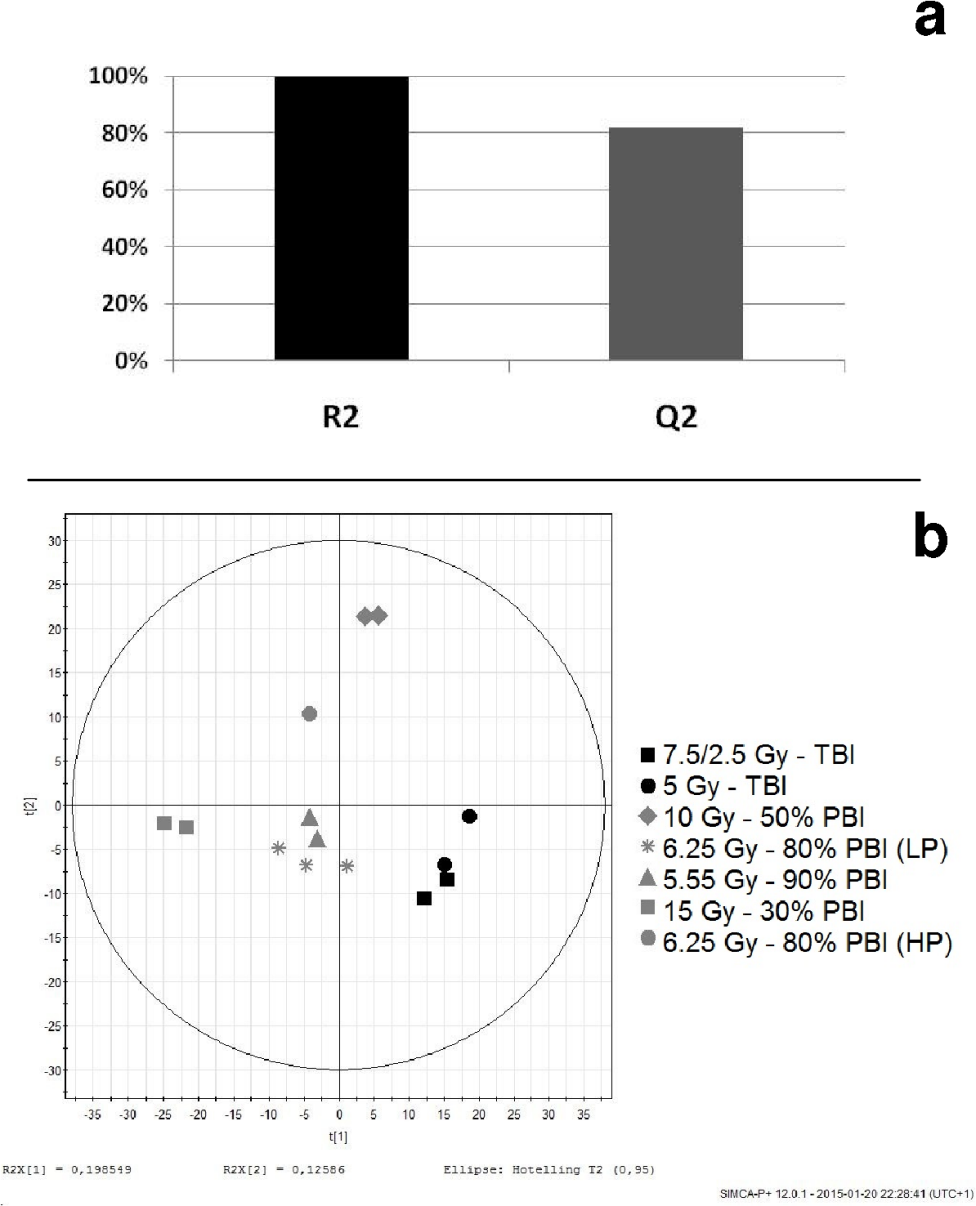
Validation of the animal exposure model using PLSDA. a) The R2 value of a given model may be used to assess its degree of fit to the data (99.8%) and the Q2 value of the model is used to assess the predictive power of the model (82.0%). b) Scores plot showing a clear distinction of 5Gy TBI animals from the 5 Gy equivalent PBI. LP = Legs Protected. HP = Head Protected.

PLSDA applied to the 14 baboons exposed to an equivalent of 5 Gy showed a very good validation of the model (Fig 1A) and a clear discrimination between TBI and PBI animals (Fig 1B).

**Fig 2:**
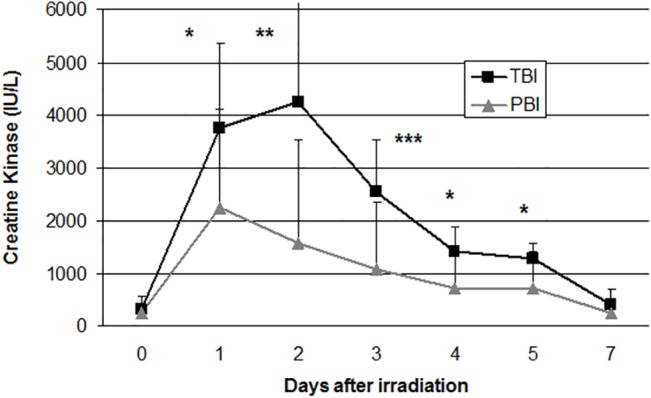
Kinetics of Creatine Kinase (CK) as an example of an early biomarker. The average of CK values of TBI animals are significantly higher than PBI animals from 1d after exposure (*p<0.05; **p<0.01; ***p<0.001).

Using a univariate logistic regression on data of pooled animals (14849 values from 6 TBI baboons and 12 PBI baboons), 23 variables (out of 925) were assessed as either significantly associated with the exposure or representing complete separation of the TBI from the PBI data set at certain time points after irradiation (Table 1). Variables that had low sample sizes per group (n≤10 for TBI and n≤4 for PBI) were excluded and 456 showed no significant association with the exposure. AST, CK, iron, Flt-3 Ligand, red blood cells, Hb, PLT and DIC were among the biomarkers identified. It can be seen in Fig 2 the kinetics of CK, as an example of an early biomarker.

**Table 1:** Descriptive statistics and odds ratio calculations using logistic regression analysis.

Variable /days after irradiation		TBI					PBI					TBI relative to PBI			
		n	mean	SD	min	max	n	mean	SD	min	max	OR	95% CI	chi²	ROC
**Blood chemistry**															
**AST (U/L)**	**1d**	6	201.8	74.3	117.0	293.0	12	85.7	61.6	16	203	1.02	1.003–1.04	0.02	0.89
**CK (U/L)**	**2d**	6	4237.3	1982.6	1743	7044	12	1570.1	1957.3	152	6720	1.001	1.000–1.002	0.04	0.86
**Iron (μM/L)**	**10d**	6	44.6	1.3	43.1	46.0	12	27.0	6.9	19	38.2			Compl.Sep.	
**Flt-3 Ligand (pg/mL)**	**14d**	6	1835.5	882.7	666.0	2702.0	12	287.9	337.3	107	1333	1.004	1.000–1.007	0.04	0.97
**Cellular blood components**															
**RBC (10e12/L)**	**21d**	6	2.9	0.5	2.0	3.3	12	4.4	0.3	3.6	4.8			Compl.Sep.	
	**24d**	6	2.8	0.7	1.5	3.6	12	4.5	0.3	3.9	4.9			Compl.Sep.	
	**28d**	5	3.2	0.2	3.0	3.6	12	4.6	0.3	3.8	4.9			Compl.Sep.	
	**35d**	5	4.0	0.2	3.7	4.3	10	4.8	0.3	4.3	5.3			Compl.Sep.	
**Leukocytes (10e09/L)**	**24d**	6	1.0	0.5	0.2	1.7	12	4.2	1.6	1.9	6.6			Compl.Sep.	
**Hematocrite (%)**	**21d**	6	22.2	3.8	15.1	25.1	12	33.6	2.7	27.9	36.9			Compl.Sep.	
	**24d**	6	21.1	5.2	10.9	26.2	12	34.5	2.7	29.9	37.7			Compl.Sep.	
	**35d**	5	30.5	1.4	28.7	32.4	10	36.9	2.4	33.6	41.2			Compl.Sep.	
**Hb (g/dL)**	**14d**	6	9.2	0.7	8.3	10.3	12	10.9	1.1	8.9	12.8	0.11	0.02–0.82	0.03	0.92
	**21d**	6	7.2	1.2	5.0	8.1	12	11.2	0.8	9.4	12.1			Compl.Sep.	
	**24d**	6	6.9	1.9	3.4	9.0	12	11.5	0.8	10.1	12.3			Compl.Sep.	
	**28d**	6	8.1	0.6	7.5	9.0	12	11.6	0.7	9.9	12.5			Compl.Sep.	
	**35d**	5	10.0	0.4	9.4	10.5	10	12.2	0.5	11.5	12.9			Compl.Sep.	
**MONO (10e09/L)**	**17d**	6	0.03	0.01	0.02	0.05	12	0.2	0.1	0.1	0.5			Compl.Sep.	
**NLR**	**24d**	6	0.5	0.8	0.1	2.0	12	1.8	1.2	0.6	4.8	0.104	0.01–0.89	0.04	
**PLT (10e09/L)**	**14d**	6	10.8	5.6	6.0	20.0	12	144.7	90.8	57	372			Compl.Sep.	
	**17d**	6	10.5	8.0	1.0	22.0	12	149.3	46.4	81	245			Compl.Sep.	
**Cytogenetics**															
**DIC frequency**	**1d**	6	0.9	0.4	0.3	1.2	12	0.4	0.3	0.03	0.97	29.3	1.04–824.6	0.05	0.80

Twenty three parameters either significantly associated or showing complete separation of TBI versus PBI data sets at an equivalent dose of 5 Gy or 2.5 Gy TBI.

SD = Standard Deviation; Compl.Sep. = Complete Separation; CI = Confidence Interval; chi^2^ = chi-square p-value.

**Fig 3:**
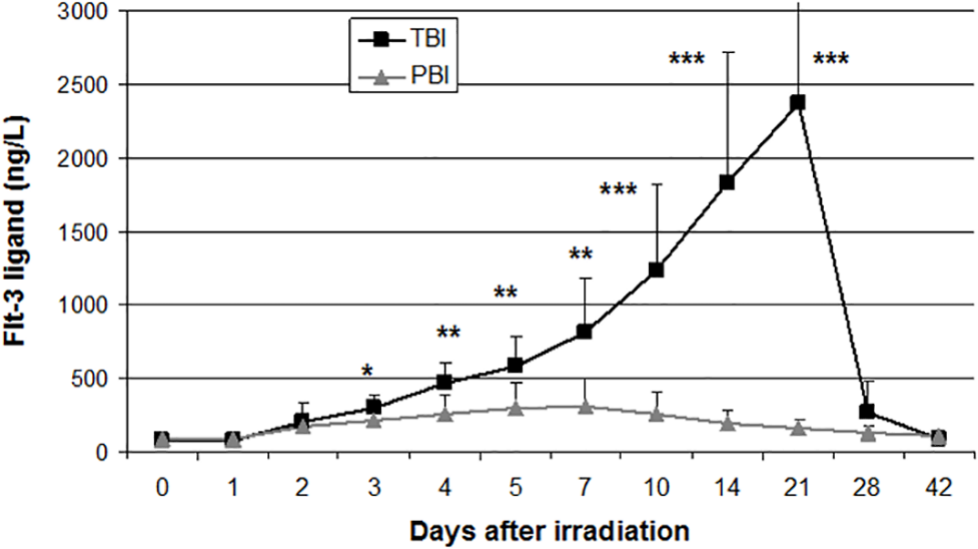
Flt-3 ligand kinetics as an example of biomarker with long time window of relevance. The averaged values of TBI animals are significantly higher than PBI animals from 3d after exposure (*p<0.05) and peak 21d post-exposure (**p<0.01 and ***p<0.001).

Concerning the Mann-Whitney test, the same parameters were identified for longer time windows. More precisely the comparison of mean values from 9 plasmatic biochemical markers and most of hematological parameters appeared significantly different between groups of baboons that received either a PBI or a TBI during the prodromal phase (0.25d–7d) and the manifest illness phase (10d–28d). The most significant biomarkers of exposure for the early period were AST, CK, LDH, urea, EPO and coagulation factor V (Table 2). The most significant biomarkers of exposure for the delayed phase were Hb, Ly, PLT and MONO (Table 3). Moreover, ANC, CRP, Iron, Flt-3 Ligand and NLR showed a long window of relevance for identification of TBI versus PBI (Table 4, and Fig 3 for Flt-3 Ligand specifically) which covers both early days and the manifest illness phase (up to 28d). Later on two hematologic parameters (Hb up to 42d and PLT up to 90d) still showed significant differences between PBI and TBI groups of animals. Interestingly, the results presented here were independent of pre-irradiation values.

**Table 2:** Biomarkers useful for early identification of TBI versus PBI.

Biomarker	Day after irradiation	TBI group (Mean ±sem)	PBI group (Mean ±sem)	Statistical significance (p)
**AST (IU/L)**	before	23± 7	20.6 ± 9.7	ns
	1	201.8± 74.3	85.7± 61.6	< 0.01
	2	133.3 ± 65.4	73.1± 62.9	< 0.05
	3	113.3± 41.1	55.3± 43.8	< 0.025
	4	76.7± 25.8	44.3 ± 32.7	< 0.025
	5	63.2± 17.2	40± 25	< 0.05
**CK (IU/L)**	before	323.7± 240.6	239± 127	ns
	1	3744± 1608	2255± 1855	< 0.05
	2	4237± 1983	1570±1957	< 0.01
	3	2537± 979	1070± 1299	< 0.001
	4	1416± 470	717± 743	< 0.05
	5	1271 ± 310	729± 681	< 0.05
**EPO (IU/L)**	before	3± 1.3	3.6± 4.8	ns
	7	22.5± 8.5	9.8± 7.9	< 0.01
**Factor V (%)**	before	75.8± 21.1	68.1± 15.8	ns
	7	100.7± 19.5	82.8 ± 26.5	< 0.025
**LDH (IU/L)**	Before	355 ±84	358± 104	ns
	2	1164± 456	774± 555	< 0.025
	3	931 ± 321	714 ± 386	< 0.05
	4	764± 189	467± 208	< 0.01
	5	689 ± 190	543 ± 165	< 0.05
**Urea (mM/L)**	Before	4.4 ± 0.3	4 ± 0.8	ns
	1	9.8± 0.6	7.3± 2.3	< 0.025
	2	9.7 ± 1.6	5.9± 2	< 0.01
	3	6.9± 1.4	5.2± 1.3	< 0.01

TBI (n = 6) PBI (n = 12)

**Table 3:** Biomarkers useful for delayed identification of TBI versus PBI.

Biomarker	Day after irradiation	TBI group (Mean ±sem)	PBI group (Mean ±sem)	Statistical significance (p)
**Hemoglobin (g/dL)**	before	13.3± 0.5	13.2 ± 0.9	ns
	10	10.1± 1	11.2± 1	< 0.05
	14	9.2± 0.7	10.9± 1.1	< 0.01
	17	8.4± 0.7	11.1± 1	< 0.001
	21	7.2± 1.2	11.2± 0.8	< 0.001
	24	6.9± 1.9	11.5± 0.8	< 0.001
	28	8.1± 0.6	11.6 ± 0.7	< 0.001
	35	10± 0.4	12.2± 0.5	< 0.01
	42	10.8± 1	12.4 ± 0.6	< 0.01
**ALC (10e9/L)**	before	2.4 ± 1.5	3.1 ±1	ns
	10	0.34 ± 0.14	0.92 ± 0.82	< 0.025
	14	0.39± 0.31	0.95± 0.62	< 0.01
	17	0.52± 0.18	1.38±0.93	< 0.01
	21	0.61 ± 0.48	1.39 ± 1.03	< 0.025
	24	0.63 ± 0.34	1.45 ± 0.6	< 0.01
**MONO (10e9/L)**	before	0.25 ± 0.13	0.25 ± 0.08	ns
	14	0.04 ± 0.04	0.16 ± 0.07	< 0.01
	17	0.03 ± 0.01	0.23 ± 0.13	< 0.001
	21	0.03 ± 0.02	0.19 ± 0.08	< 0.001
**Platelets (10e9/L)**	before	213.8± 72.3	255.3±87.2	ns
	10	66.8± 26.5	169.8± 111.7	< 0.01
	14	10.8± 5.6	144.7± 90.8	< 0.001
	17	10.5± 8	149.3± 46.4	< 0.001
	21	69.5± 65.1	175.9± 51.9	< 0.01
	60	140.6 ± 55.5	212.4 ± 68.4	< 0.01
	90	146.8 ± 45.2	196 ± 54.5	< 0.05

TBI (n = 6) PBI (n = 12)

**Table 4:** Biomarkers with a long window of relevance for identification of TBI versus PBI.

Biomarker	Day after irradiation	TBI group (Mean ±sem)	PBI group (Mean ±sem)	Statistical significance (p)
**ANC (10e9/L)**	Before	4.7± 2.7	3.9± 1.8	ns
	0.25	19± 2	14.9± 3.4	< 0.025
	1	10.7±2.1	7.7± 2.9	< 0.05
	10	1± 0.5	2± 1.5	< 0.05
	21	0.69± 1.2	2.2 ± 1.2	< 0.01
	24	0.23 ± 0.35	2.4± 1.2	< 0.001
**CRP (mg/L)**	before	6.7 ± 2.4	5.5± 3.2	ns
	5	19.6± 9.9	10.4± 7	< 0.025
	7	17.9± 10.4	6.5 ± 3.3	< 0.025
	28	40.5± 39.2	5.4± 3.5	< 0.01
**Iron (mcM/L)**	before	29.5± 5	30.7± 3.8	ns
	5	48.6± 3	30.6± 10.9	< 0.01
	7	45.9± 3	29.6± 8.6	< 0.01
	10	44.9± 1.1	27± 6.9	< 0.001
	14	42.4± 9.5	24± 3.9	< 0.001
	21	43.2± 5.3	24 ± 5.9	< 0.001
**Flt-3 Ligand (ng/L)**	before	75.3± 36	84.3± 22.6	ns
	3	293.5 ± 88.8	209.8 ± 90.5	< 0.05
	4	463.5± 134.9	251.8± 129.5	< 0.01
	5	583.2± 197.9	290.5± 168.1	< 0.01
	7	811.5± 366.7	307.7±191.2	< 0.01
	10	1230± 589.6	254.7± 147.7	< 0.001
	14	1835± 883	192.8± 77.3	< 0.001
	21	2374± 885	160.3± 46.4	< 0.001
**NLR**	0	2.5 ± 1.8	1.4 ± 0.5	ns
	0.5	21.7 ± 6.3	16.4 ± 4.7	< 0.05
	1	26.4 ± 7.3	17.6 ± 7.1	< 0.025
	21	1.3 ± 2	1.8 ± 1	< 0.05
	24	0.5 ± 0.8	1.8 ± 1.2	< 0.01
	28	1 ± 0.8	1.8 ± 0.9	< 0.05

TBI (n = 6) PBI (n = 12)

For the DIC assay specifically the results were analysed per pair of animals as this marker is currently the reference for the classification of exposure victims. The u-values were most effective at classifying partial irradiations 1d after exposure: 92%. The average estimations for this time are shown in Table 5. At later times (28 and 200 days for this study), most PBI are still detected but false positives (TBI incorrectly classified as PBI) begin to appear.

**Table 5:** DIC assay estimations of the percentage of circulating blood exposed from a sample taken 1 day after exposure.

Fraction of BM exposed	100%	50%	100%	100%	90%	80% (LP)	50%	30%	80% (HP)
**Dose (Gy)**	**2.5**	**5**	**5**	**7.5/2.5**	**5.55**	**6.25**	**10**	**15**	**6.25**
**Estimated fraction of blood exposed**	100%	88%	100%	100%	93%	83%	70%	20%	55%
**Estimated Dose (Gy)**	2.5	3.2	4.7	4.7	4.9	5.4	4.6	3.7	4.8

LP = Legs Protected. HP = Head Protected.

**Fig 4:**
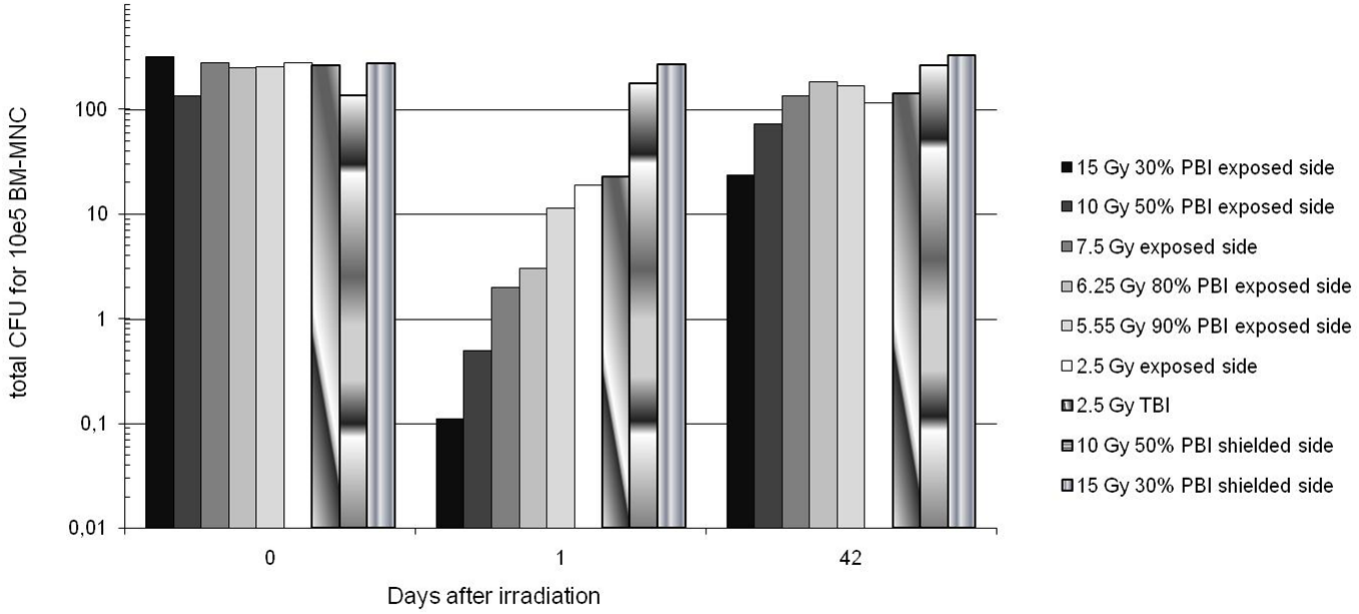
Hematopoietic bone marrow (BM) colony-forming units from exposed and shielded humerus. The values here represented are the average of the 2 animals of each exposure group. The exposed areas showed a dose-dependent decrease 1d after exposure and there’s a start of recovery at 42d. In the legend “10 Gy 50% PBI exposed side” refers to bone marrow cells extracted from the exposed side of the animal subjected to a 10 Gy 50% PBI. “10 Gy 50% PBI shielded side” refers to bone marrow cells extracted from the shielded side of the animal (shielded using a 20 cm thick lead screen) subjected to a 10 Gy 50% PBI.

The numbers of BM cells capable of dividing (all lineages confounded) in the exposed areas showed a dose-dependent decrease 1d after exposure. A start of recovery could be seen at 42d (Fig 4).

To assess the impact of frequent blood sampling on hematologic status, a group of three age-matched baboons was used as sham-irradiated individuals. Twenty-one days after sham-irradiation, red blood cells and haemoglobin levels were in average 96% and 99% respectively those of base line values.

## Discussion

Predicting the damage undergone by individuals externally exposed to ionizing radiation following an unexpected nuclear or radiological event is a complex issue. An event like this would lead to a diverse injured population, with different radiation doses and degrees of bone marrow shielding. This diversity was reproduced here by the inclusion of pairs of baboons with different dose-equivalent exposures in two groups, TBI and PBI, which are compared. The baboon model was chosen for its genetic and physiological proximity to humans and its size, which allowed us to take in consideration the impact of corpulence in dose distribution.

Radiation injury might not be an emergency in comparison with blast, but haemorrhage and trauma, exposure to severe radiation doses and/or combined injuries still require rapid clinical evaluation with estimation of both radiation dose and heterogeneity [13]. Indeed, in addition to triaging “worried well” and irradiated victims, medical responders have to distinguish PBI and TBI to identify individuals needing hematologic support.

The goal was to identify biomarkers of exposure that could contribute to patients’ diagnosis and prognosis using deployable biodosimetry tools. We therefore evaluated in baboons the capacity of a few biomarkers to discriminate between different situations of TBI and PBI equivalent to a global exposure in the range of 2.5 Gy to 5 Gy which accounts for documented radiation accidents. Indeed baboons exposed to TBI from 2 Gy develop a classical acute radiation syndrome [8] close to what is observed in humans. Slightly less radiosensitive than humans, baboons show a LD50/60 following unilateral gamma irradiation in the 6 Gy to 7 Gy range. In previous publications, we validated the baboon as an adequate radiation exposure model to study TBI versus PBI [8] and focused on the first 7 days after exposure 5 Gy equivalent exposures [9].The present study is far more complete in terms of studied parameters, statistical strategy and radiation doses.

The analysis was based on three independent statistical approaches. A first general analysis method was PLSDA and it was carried out on all the data of all 5 Gy equivalent baboons. The fact that TBI baboons results were similar (plotted together) and well separated from PBI validated a model that intended to discriminate partial from total exposures. In the second (univariate logistic regression) and third (Mann-Whitney) approaches, the analysis of each biomarker was done by comparing the combined PBI data with the combined TBI data.

Here, two baboons required irradiated blood cell transfusions, one animal exposed to 5 Gy TBI and the other to 7.5 Gy / 2.5 Gy TBI. The latter was euthanized on day 25 because of an irreversible bone marrow aplasia. Thus, the course of exploration in this study covered not only the period of diagnosis but also the manifest illness phase when patients’ clinical review could be required for treatment decision making. Furthermore, the assessment of two different equivalent doses was aimed at testing the robustness of the identified biomarkers to distinguish TBI and PBI.

Although a relevant early bio-indicator of exposure for TBI [4–6], CRP did not prove in our hands to discriminate PBI and TBI until after day 5 [9]. CRP has to be carefully taken into account because this is a non specific inflammatory marker sensitive to many factors (trauma, stress, infection…). Furthermore, CRP levels also increased significantly in TBI animals only during hematopoietic recovery, to higher values than those observed over the radiation-induced acute phase.

Not surprisingly hematologic parameters were able to make the difference between our models of TBI and PBI, as the latter were mostly designed in terms of percentage of BM sparing. Thus ANC, ALC, Hb, MONO, PLT and more especially Flt3-ligand as an early predictor [14], were strong indicators of BM aplasia and could therefore help make a decision as to provide hematologic support. In this context, Flt3-ligand appeared particularly robust to separate situations such as TBI and 90% PBI. Previous studies already showed that the amount of Flt3-ligand in the blood of patients during local radiotherapy correlated with the proportion of irradiated bone marrow [15]. Recently, it has been shown in mice 1d and 2d after TBI or PBI that increases in the body fraction exposed induced progressive decreases in lymphocyte counts and increases in the NLR with no significant differences in the neutrophil and platelet counts [16]. Moreover, the radioresponse for plasma biomarker Flt3-ligand showed proportional increases.

Iron level is also related to hematopoietic tissue response and the TBI group showed from 5d to 21d a durable increase that may be linked to intravascular hemolysis. Hb level decreased by 8% on average in both groups between 2d and 5d which is a common feature of radiation-induced silent hemolysis. Moreover, preliminary data suggested that EPO plasmatic levels could be a usable biomarker from 7d or even more 10d after irradiation, the greater the volume of exposed body the higher the EPO level.

Different enzymes such as AST, LDH and CK, known markers of tissue injury, rose greatly early after exposure with higher levels following TBI. AST increased whereas ALT did not which may account for general visceral injury affecting the heart, the liver, skeletal muscle and kidneys. The great content of LDH in RBC could account for hemolysis, preceding iron increase. CK exhibited the strongest increase very probably in connection with skeletal muscle disorder in inflammatory context. Urea increased in both irradiation situations but greater after TBI reflecting a transient kidney dysfunction justifying a mechanistic study.

Additional parameters that would be easy to deploy (i.e rapid, automated, inexpensive) such as the PB and reticulocyte count [17] could be added to improve the evaluation of heterogeneous accidental irradiations. More sophisticated markers such as PB CD34+ cells (requiring flow cytometry) could also be interesting as their level is known to change quickly after exposure to ionizing radiation. To continue the research here presented, other early biomarkers must be considered. Citrulline, for instance, despite being more expensive and difficult to deploy, might prove helpful to anticipate the management of acute gastro-intestinal (GI) syndrome [18]. However, in this study, citrulline levels did not appear relevant for distinguishing TBI vs PBI.

This study strongly suggests that among the numerous parameters investigated in baboons irradiated within the range of 2.5 to 5 Gy, several bio-markers are capable of distinguishing the type of exposure at least within the dose range studied.

In practice, in case of a large size NR event, a consensus radiation categorization such as METREPOL (Medical Treatment Protocols for Radiation Accident) would be employed as it provides early diagnosis and clinical re-evaluation of irradiated patients based on clinical signs and symptoms, and lymphocyte count kinetics [19]. The parameters evaluated in this study need to be confronted by analysing history clinical cases of irradiation. Validated biomarkers could then be included in a diagnostic and prognostic approach to provide irradiated casualties with better medical care.

## References

[1] Hérodin F, Drouet M. Cytokine-based treatment of accidentally irradiated victims and new approaches. ExpHematol. 2005 10;33(10):1071–80. 10.1016/j.exphem.2005.04.007 16219528

[2] Trott KR, van Luijk P, Ottolenghi A, Smyth V. Biological mechanisms of normal tissue damage: importance for the design of NTCP models. RadiotherOncol. 2012 10;105(1):79–85. 10.1016/j.radonc.2012.05.008. Epub 2012 Jun 29 22748390

[3] Prasanna PGS, Moroni M Pellmar TC. Triage dose assessment for partial-body exposure: dicentric analysis. Health Phys. 2010 2;98(2):244–51. 10.1097/01.HP.0000348020.14969.4 20065689 PMC2806648

[4] Ossetrova NI, Blakely WF. Multiple blood-proteins approach for early-response exposure assessment using an in vivo murine radiation model. Int J Radiat Biol. 2009;85(10):837–50. 19863200

[5] Ossetrova NI. Sandgren DJ, Blakely WF. C-reactive protein and serum amyloid A as early-phase and prognostic indicators of acute radiation exposure in nonhuman primate total-body irradiation model. Radiat Maes. 2011; 46:1019–1024;. 10.1016/j.radmeas.2011.05.021

[6] Blakely WF, Ossetrova NI, Whitnall MH, Sandgren DJ, Krivokrysenko VI, Shakhov A et al Multiple parameter radiation injury assessment using a nonhuman primate radiation model–Biodosimetry applications. Health Phys. 2010 2;98(2):153–9. 10.1097/HP.0b013e3181b0306d 20065677

[7] Hérodin F, Mestries JC, Janodet D, Martin S, Mathieu J, Gascon MP, et al Recombinant glycosylated human interleukin-6 accelerates peripheral blood platelet count recovery in radiation-induced bone marrow depression in baboons. Blood. 1992 8 1;80(3):688–95. 1638022

[8] Hérodin F, Richard S, Grenier N, Arvers P, Gérome P, Baugé S, et al Assessment of total- and partial-body irradiation in a baboon model: preliminary results of a kinetic study including clinical, physical and biological parameters. Health Phys. 2012 8;103(2):143–9. 10.1097/HP.0b013e3182475e54 22951472

[9] Hérodin F, Valente M, Abend M. Useful radiation dose biomarkers for identification of early partial-body exposures. Health Phys. 2014 6;106(6):750–4. 10.1097/HP.0000000000000059 24776909

[10] Norol F, Drouet M, Mathieu J, Debili N, Jouault H, Grenier N, et al Ex vivo expanded mobilized peripheral blood CD34+ cells accelerate hematologic recovery in a baboon model of autologous transplantation. Br J Haematol. 2000 4; 108:1–12. 10.1046/j.1365-2141.2000.01995.x 10848796

[11] Dolphin GW. Biological dosimetry with particular reference to chromosome aberration analysis. A review of methods. IAEA, Vienna, Vol. Handling of Radiation Accidents, 215–224; 1969

[12] Deperas J, Szluinska M, Deperas-Kaminska M, Edwards A, Lloyd D, Lindholm C, et al CABAS—a freely available PC program for fitting calibration curves in chromosome aberration dosimetry. RadiatProt Dosimetry. 2007;124(2):115–23. 10.1093/rpd/ncm137 18073230

[13] Rojas Palma C. [edit.], e.a.- TMT Handbook—Triage, Monitoring and Treatment of people exposed to ionising radiation following a malevolent act.- Osteräs, Norway: NRPA, 2009.- 556 p.- ISBN 978-82-90362-27-5.

[14] Bertho JM, Demarquay C, Frick J, Joubert C, Arenales S, Jacquet N, et al Level of Flt3-ligand in plasma: a possible new bio-indicator for radiation-induced aplasia. Int J Radiat Biol. 2001 6;77(6):703–12. 11403710 10.1080/09553000110043711

[15] Huchet A, Belkacémi Y, Frick J, Prat M, Muresan-Kloos I, Altan D, et al Plasma Flt-3 ligand concentration correlated with radiation-induced bone marrow damage during local fractionated radiotherapy. Int J RadiatOncolBiol Phys. 2003 10 1;57(2):508–15. 10.1016/s0360-3016(03)00584-4 12957264

[16] Blakely WF, Sandgren DJ, Nagy V, Kim SY, Sigal GB, Ossetrova NI. Further Biodosimetry Investigations Using Murine Partial-Body Irradiation Model. RadiatProt Dosimetry. 2014 6;159(1–4):46–51. 10.1093/rpd/ncu127 24757174

[17] Chaudhuri JP, Metzger E, Messerschmidt O. Peripheral reticulocyte count as biologic dosimetry of ionizing radiation. Experiments in the mouse. ActaRadiolOncolRadiatPhys Biol. 1979;18(2):155–60. 10.3109/02841867909128202 495191

[18] Prasanna PGS, Blakely WF, Bertho JM, Chute JP, Cohen EP, Goans RE, et al Synopsis of partial-body radiation diagnostic biomarkers and medical management of radiation injury workshop. Radiat Res. 2010 2;173(2):245–53. 10.1667/RR1993.1 20095857 PMC8914528

[19] Friesecke I, Beyrer K, Fliedner TM, METREPOL team. Medical treatment protocols for radiation accident victims as a basis for a computerised guidance system. How to cope with radiation accidents: the medical management. Br J Radiol. 2001 2;74(878):121–2. 11718381 10.1259/bjr.74.878.740121

